# Supplementary material for: Bacteriocins Revitalize Non-Effective Penicillin G to Overcome Methicillin-Resistant Staphylococcus pseudintermedius
Source: Antibiotics (Basel). 2022 Nov 24;11(12):1691. doi: 10.3390/antibiotics11121691 (PMC9774949; doi:10.3390/antibiotics11121691)
Supplement: Supplementary file 1 [file antibiotics-11-01691-s001.zip › antibiotics-2043156-supplementary.pdf]

Supplementary materials

Supplemental Table 1. Antibiotic resistance profile of MRSP isolates from dog skin infection. S – sensitive, R – resistant. The stars (*) indicate resistant mutants appearing inside inhibition zone if incubated longer than 24h.

	Isolate / Antibiotic	LMGT 4218	LMGT 4219	LMGT 4220	LMGT 4221	LMGT 4222	LMGT 4223
1	Ceftriaxone	R	R	R	S	S	R
2	Chloramphenicol	S	S	S	S	S	S
3	Ciprofloxacin	S	R	R	S	S	S
4	Clindamycin	S	R	S	S	S	R
5	Cloxacillin	R	R	R	R	R	R
6	Erythromycin	S	R	S	S	S	R
7	Fusidic acid	S*	S*	R	S*	S*	S*
8	Gentamicin	R	R	S	S	S	R
9	Kanamycin	R	R	R	R	R	R
10	Ofloxacin	S	R	R	S	S	S
11	Penicillin G	R	R	R	R	R	R
12	Streptomycin	R	R	R	R	R	R
13	Tetracycline	R	R	S	R	R	R
14	Trimethoprim	R	R	S	R	S	R

See Materials and Methods section for detail of the assay and the conditions used.

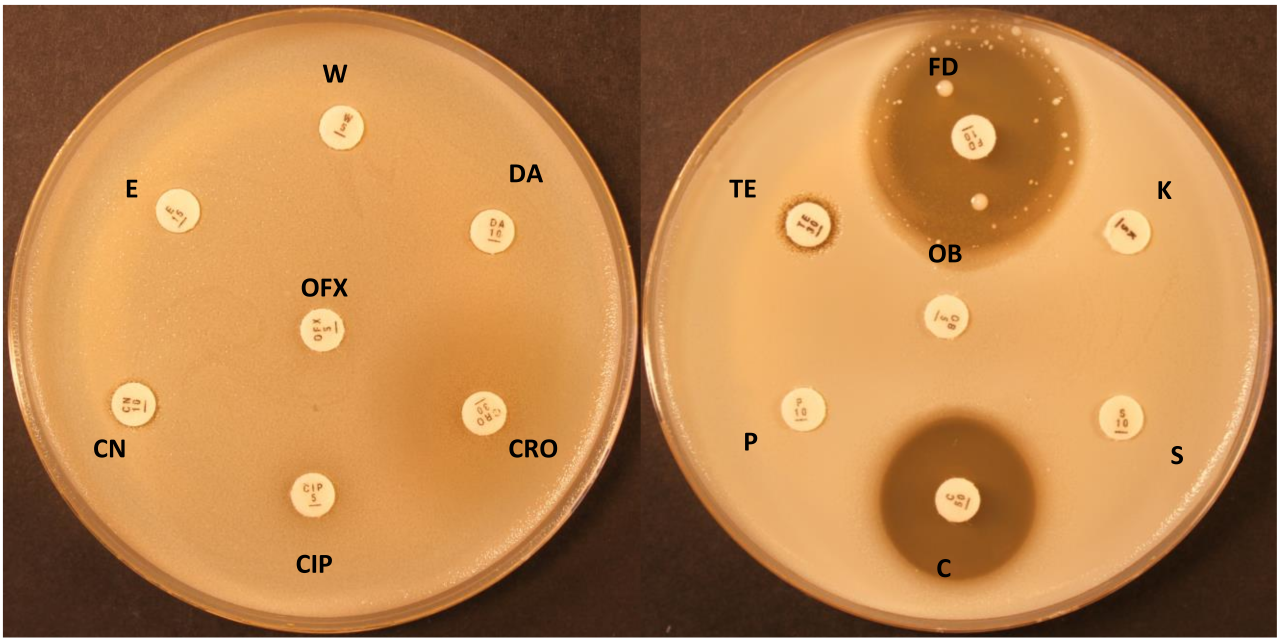

Supplemental Figure 1

Antibiotic resistance profile of MRSP LMGT 4219

Antibiotic discs – FD-fusidic acid, 5 µg; K – kanamycin, 5 µg; S – streptomycin, 10µg; C – chloramphenicol, 50µg; P – penicillin G – 10µg; TE – tetracycline, 30µg; OB – cloxacillin, 5µg; W – trimethoprim, 5µg; DA – clindamycin, 10µg; CRO – ceftriaxone, 30µg; CIP – ciprofloxacin, 5µg; CN – gentamicin, 10µg; E – erythromycin, 15µg; OFX – ofloxacin, 5µg. Note that many white colonies appear within the inhibition zone of fusidic acid (FD), a typical sign of resistance development which has also been reported for *S. aureus* [21].

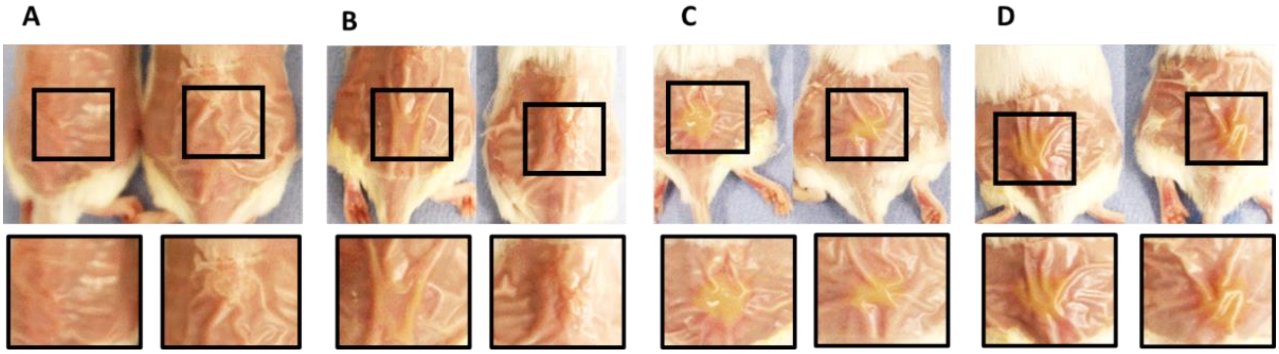

Supplemental Figure 2

Mouse wounds 24 h post infection with different inoculum size of LMGT 4219

A - 0 CFU; B – 10^6 CFU; C – 10^7 CFU; D – 10^8 CFU. Infected sites are magnified in the lower panel.

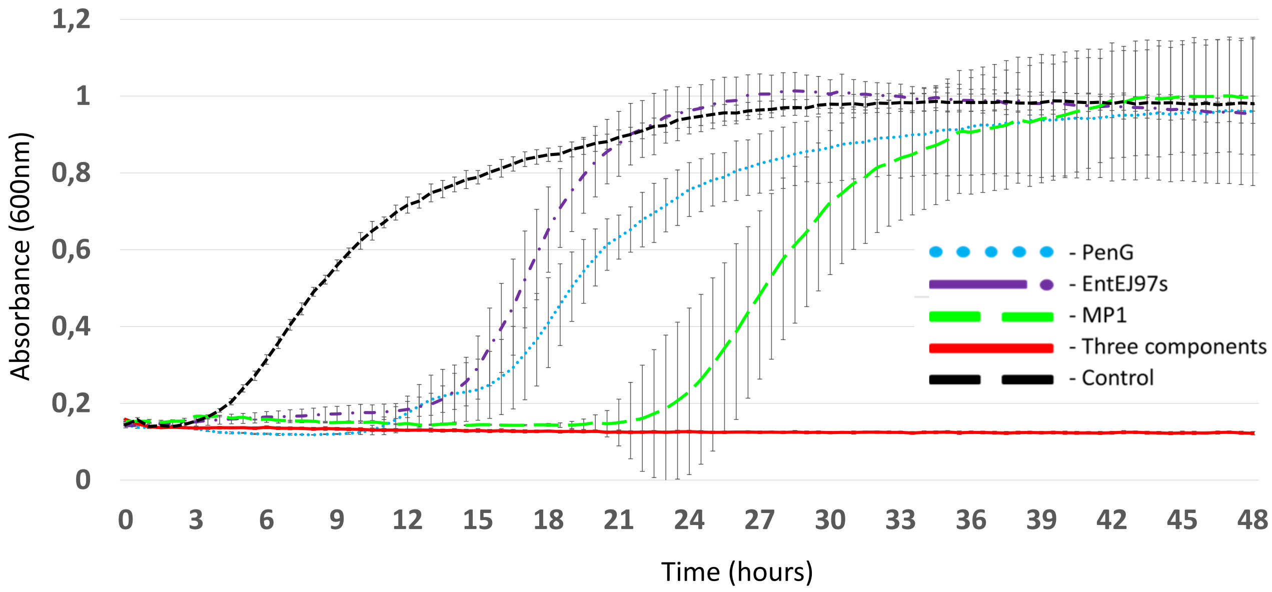

Supplemental Figure 3

Growth curves of LMGT 4219 in the presence of different antimicrobials: PenG (250 µg/ml), EntEJ97s (50 µg/ml), MP1 (10 µg/ml), and the combination of all three antimicrobials.
